# False–positive phenomenon in microbubble testing: unveiling the underlying mechanism

**DOI:** 10.1093/ehjimp/qyaf056

**Published:** 2025-05-28

**Authors:** Yumi Yamamoto, Yasuhide Mochizuki, Saaya Ichikawa-Ogura, Rumi Hachiya, Hiroto Fukuoka, Toshiro Shinke

**Affiliations:** Division of Cardiology, Department of Medicine, Showa Medical University School of Medicine - Hatanodai Campus, 1-5-8 Hatanodai Shinagawa-ku, Tokyo 142-8555, Japan; Division of Cardiology, Department of Medicine, Showa Medical University School of Medicine - Hatanodai Campus, 1-5-8 Hatanodai Shinagawa-ku, Tokyo 142-8555, Japan; Division of Cardiology, Department of Medicine, Showa Medical University School of Medicine - Hatanodai Campus, 1-5-8 Hatanodai Shinagawa-ku, Tokyo 142-8555, Japan; Division of Cardiology, Department of Medicine, Showa Medical University School of Medicine - Hatanodai Campus, 1-5-8 Hatanodai Shinagawa-ku, Tokyo 142-8555, Japan; Division of Cardiology, Department of Medicine, Showa Medical University School of Medicine - Hatanodai Campus, 1-5-8 Hatanodai Shinagawa-ku, Tokyo 142-8555, Japan; Division of Cardiology, Department of Medicine, Showa Medical University School of Medicine - Hatanodai Campus, 1-5-8 Hatanodai Shinagawa-ku, Tokyo 142-8555, Japan

**Keywords:** echocardiography, patent foramen ovale, valsalva manoeuvre, microbubble test, non-smoke spontaneous individual contrast

We present two interesting cases of false–negative findings in microbubble tests. A woman in her 50 s (Patient 1), diagnosed with cerebral embolism in the middle cerebral artery, underwent transoesophageal echocardiography (TOE) to investigate embolic stroke of undetermined source. A microbubble test using agitated saline on TOE under the awake state suggested a right-to-left shunt (RLS) of Grade III during the Valsalva manoeuvre (VM) without external abdominal compression, indicating paradoxical embolism due to a patent foramen ovale (PFO). However, microbubble tests using both transthoracic echocardiography and transcranial Doppler were negative for RLS. Careful observation of the TOE revealed that microbubble-like echoes suddenly appeared in the left atrium (LA) within three cardiac cycles immediately after VM release, showing filamentous, high echogenicity resembling a snowstorm without PFO (*Figure 1A*, Supplementary data online, *Video S1*). This phenomenon was identified as non-smoke spontaneous individual contrast (NSSIC), characterized by rouleaux formation of blood in the pulmonary veins and appearing in the LA during VM.

**Figure 1:**
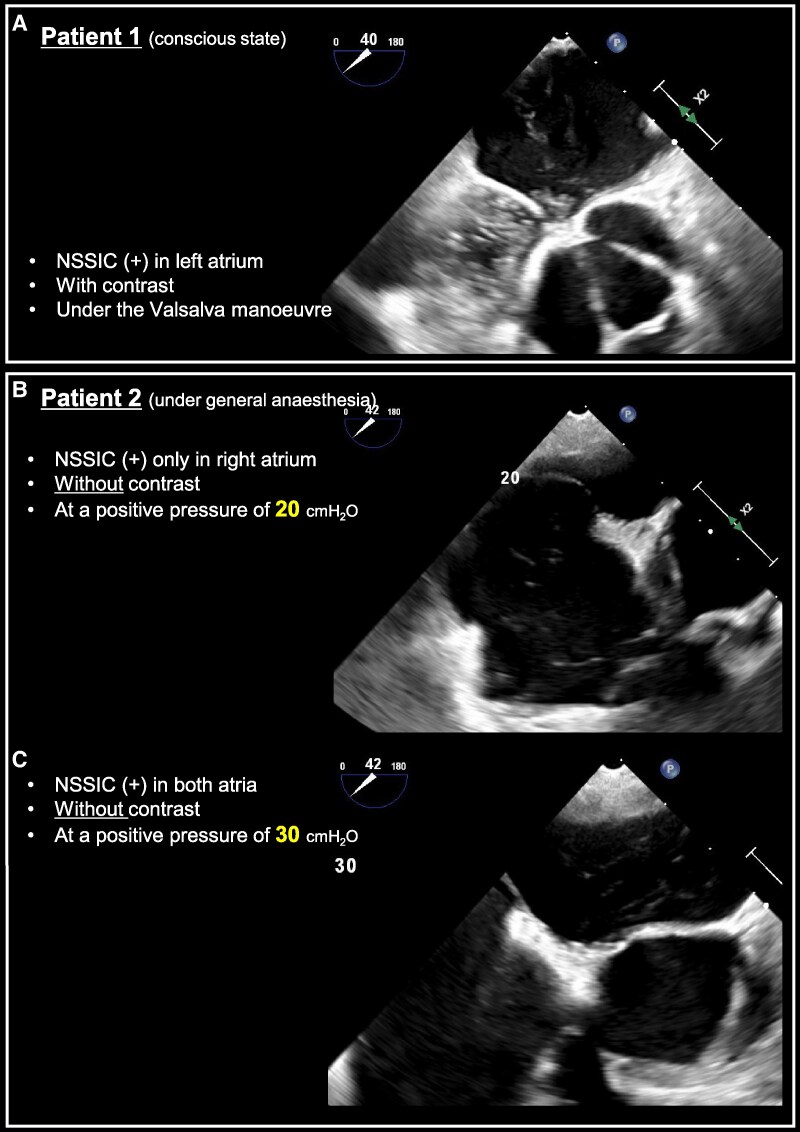
A NSSIC-positive case during microbubble testing in TOE without PFO (*A*). (*B* and *C*) TOE performed under general anaesthesia in a patient undergoing percutaneous PFO closure, using 10 s of positive pressure ventilation without contrast bubbles. In *B*, positive pressure ventilation at 20 cmH₂O induced NSSIC in the right atrium only, whereas in *C*, ventilation at 30 cmH₂O resulted in NSSIC being observed in both the right and left atria. NSSIC, non-smoke spontaneous individual contrast; TOE, transoesophageal echocardiography; PFO, patent foramen ovale.

In another patient (Patient 2: a man in his 50 s) with PFO, when VM was applied via positive pressure ventilation under general anaesthesia during percutaneous PFO closure, the same phenomenon occurred without microbubble injection. Interestingly, at a positive pressure ventilation of 20 cmH_2_O, this phenomenon was observed only in the right atrium (*Figure 1B*, Supplementary data online, *Video S2*), while at 30 cmH_2_O, it appeared in both atria (*Figure 1C*, Supplementary data online, *Video S3*). These cases suggest that the intensity of VM may influence the appearance of NSSIC. Patients capable of strong VM should be carefully evaluated for this false–positive phenomenon.

## Supplementary Material

qyaf056_Supplementary_Data

## Data Availability

The anonymized data underlying this article will be shared upon reasonable request with the corresponding authors.

## Supplementary data

**Consent:** The authors confirm that written consent for the submission and publication of this case report, including images and associated text, was obtained from the patient in line with COPE guidelines.

## Lead author biography

Dr Yumi Yamamoto graduated from the Faculty of Medicine at Chiba University, Japan in March 2020. Since April 2022, she has been working in the Department of Cardiology at Showa Medical University Hospital in Tokyo. She specializes in cardiac ultrasound and has extensive expertise in valvular heart disease, structural heart disease interventions, cardiomyopathy, and heart failure.

